# Valedictory Address

**Published:** 1862-04

**Authors:** J. H. Hollister

**Affiliations:** Professor of Anatomy


ARTICLE XI.

### VALEDICTORY ADDRESS

TO THE GRADUATING CLASS OF THE MEDICAL DEPARTMENT OF THE LIND UNIVERSITY—MARCH 4, 1862.

By J. H. HOLLISTER, M. D., Prof. of Anatomy.

GENTLEMEN:—In an age of activities, such as were never before witnessed, you are summoned to positions of manhood and the responsibilities of a noble profession. "Excelsior," is the representative motto for the men of the nineteenth century. The great mass of mind was never so thoroughly roused, so nerved to action, nor so restive in restraint as now. The attainments of yesterday, are the means of greater achievements to-day, and these give promise of a still more hopeful to-morrow. In all the avenues of trade, in the rapid development of the arts and sciences, in the advances of the professions, as in the diplomacy of nations, we may every where behold the newly wrought, but enduring monuments of masterly mental achievement.

The dark and dreamy ages, when canonized authors and antiquated theories were safe from criticism, are past. Then, the traditions of the fathers were a law unto their children for belief and action; they were innocent of departures from, or additions to the ancient faith, and never guilty of witty inventions.

But upon that Cimmerian darkness, the day began at length to dawn. For three centuries, its brightness has been ever increasing, till now it seems hastening on to its meridian splendor.

Scanning, impartially as we may, the history of the past, our wonder is that for more than fifty centuries, men could have lived and done so little. Our equal wonder is, that in the last three hundred years, they should have done so much.

The more we dwell upon the facts, the greater seems the disparity of attainments in the two periods, and the less our partiality for the present age.

Three centuries ago, the cumbrous folds of papyrus and parchment manuscripts, were the treasures of the affluent alone. To-day, the inspired utterance has its fulfillment, that to the making of books, there is no end; and the printed pages fall around us in numbers, as fall the forest leaves in autumn. They hold in sacred trust for the ages coming, the grand old thoughts quarried by master minds of former generations. They treasure alike the utterances of sympathy and selfishness, of love and hate, of gladness and sadness, that may be breathed from heart to heart.

The history of the *press* is brief. It was a mighty power unheralded. Silently moulding the opinions of mankind, it was the grand preparative for the astonishing results which we now behold. It is to-day, the grand Archimedian lever, which poised upon the fulcrum of eternal truth, moves the world.

Three centuries ago, we were ignorant of the power and use of steam. Till within our own time the noble steamer had never sported with the winds, nor bid defiance to the waves, in answer to a human will. Only fifty years ago, its first progenitor was termed the "Fulton Folly." Steam has given birth to the great railway system, and shall yet so cheapen and quicken the modes of travel, as that only the rich may afford the time and luxury of going on foot. It quickens into form, perfects and cheapens nearly every fabric that ministers to human want. It calls man upward from the menial exercise of his physical strength alone to a higher class of labors, which shall more develop his mental powers. It bids him earn his daily bread by the sweat of a brow that covers a thinking head.

A still greater marvel of our time, is the electric telegraph. Its missives are for the millions, and they out-speed the sun in flight. Though there be a temporary check to their Atlantic transit, yet steadily the tiny wires are spanning the Siberian hills, to reach us by the Behring Straits and our western shores.

Then will the evening telegram recount the days' events in every quarter of the globe. Then to, the nations shall be given a common language; from intimacy of relation shall spring a common sympathy; and the day be ushered, of a common brotherhood.

And when, as but yesterday, DAGUERRE, that light of France, commanded the sun to paint, and it obeyed; then was there a march of ages in the fine arts.

What pencil or brush so delicate and truthful as the sunbeam? what pallet of colors can equal the solar spectrum? Perish then, the art of embalming. Treasure no more the unsightly forms of departed friends, as in the catacombs of Alexandria and Thebes. How beautifully the pure light of heaven traces the sparkling, speaking, features of our friends, to be our cherished souvenirs when they are gone. The album shall be, henceforth, the representative of successive generations, esteemed in every household, the most precious of earthly treasures.

The stereoscopic panorama shall yet permit us, at a single sitting, to behold the beauties of Switzer and Italian scenery—Scotia's lakes and hills will be added to the beautiful views in our western world. We shall traverse whole continents in an evening. Then from Minnehaha to the Gulf, we shall behold the beautiful scenery of the Mississippi passing before us with not a city, bluff, or rivulet left out, all standing in bold relief, and to the eye so truthful, that we shall seem to gaze upon the scenes themselves.

To none shall it minister benefits more than to our own profession. If it trace the forms and features of those in health and beauty, it shall as faithfully portray the waste and deformity which diseases produce. It shall truthfully represent the most delicate dissections; and the man now lives, who, by means of the photographic anatomy, shall confer a signal benefit upon the profession.

To the medical student, not long in the future, shall be presented the illustrated catalogues of all the prominent museums of the world.

The stereoscope will familiarize him with the buildings and their compartments, with shelvings and alcoves, and with specimens as there arranged. Then with the history of each series of specimens before him, he may turn his eye to the magic glass and behold each specimen in its several attitudes, perfect in form and color, forbidden only the privilege of handling it. I ardently anticipate, and can hardly await the achievements of photography as a means of medical instruction.

Time would fail me to tell of the worlds of little things which the microscope is now revealing. It dissipates a thousand mysteries, revealing facts of most intense interest, which but for it, had never been known. It spreads before us volumes of evidences in Natural Theology, as beautiful, as wonderful, and as conclusive as may be the testimonies of the heaven above, or the earth around us.

I may not even advert to the multitude of wonderful inventions with which our age is teeming. These all bear witness to an energy and profundity of thought, of which till now, the human mind was deemed incapable.

Every department of science gives evidence of an awakened enthusiasm. What a triumph was that of mathematical calculation, determining the size and position of an undiscovered star, which should correllate the forces of gravitation. The telescope was adjusted to the predetermined range, and the stranger star appeared.

Now too, we read the "testimony of the rocks," and trace the records of the ages before man was made. Now chemistry brings forth her rich and varied contributious for the health and comfort of mankind, becoming the handmaid of manufacture, and the mother of agriculture.

If we turn to the professions, here are superior displays of talent and research. No theories are now too sacred for review—no human authorities dictate the forms of our belief. Proudly and respectfully we point to those venerable heroes whose feet were planted upon the ever enduring foundations of truth—but *theories*, be they never so ingenious and beautiful, yet wanting this central crowning element, are swept away, as flies the gossamer before the driving storm.

If from peaceful pursuits, we pass to those of war—here too, we find a record in keeping with the age. An army of our own brothers, more than half a million in numbers, are gone forth to defend the cherished bequest of our fathers. An army so intelligent, so finely equipped—and more brave, the world never saw—and every man, a volunteer!

Strike from the world's treasury the press and its products, and turn back to the simpler pursuits of life, its noble army of laborers; turn again to oblivion all the appliances of steam power, and the thousand facilities of manufacture; traverse the seas with fickle wind, and the land by post coaches, or on foot; let the foaming steed be the bearer of dispatches, and the human hand the power that shall minister to our daily wants; blot out the labors of the chemist, and the beauties of the photograph; strike down the serried ranks of gun clad infantry, and the iron ships of war, with rifled cannon and the death dealing mortar, and give in their place a mercenary and servile band of lancers and arrow men, with battering rams and wooden horses for fenced cities, and sling and hurling rocks for death-work on the plain; take from us the loom, the cotton gin, the reaper, the sewing machine, and a thousand like inventions, and you would return us to the condition in which for more than fifty centuries men lived in ignorance of their God-given powers, and lived to such little purpose.

View the present condition of the world, and tell me if it is not a matter of astonishment that in the last three centuries, and mainly in the *present*, such wondrous advancement should have attested the powers of the human mind, when nerved to its most vigorous efforts.

It is a noble age in which to live. And amid the competition of such activities, to stand up firmly in the great struggle, and hold an equality with the *successful*, yields a recompense which well repays the effort.

In this age, and with these surroundings, you have allied your life labors to the science of medicine, and are to devote your energies to the practice of the healing art.

And I have dwelt thus long upon what might seem trite and irrelevant on an occasion like this, that I might in this, my last address to you, impress still more indellibly the fact, that for your *success* in the profession, there is demanded of you a devotion to, and love for it, which other influences shall not divert; an energy that tires not, an enthusiasm even, which surmounts all obstacles, and satiates only with success. Harbor not the thought of filling second or third rate positions in your profession, but nerve to the conflict and welcome the effort that shall place you in the fore front of your calling.

Command, as your just desert, the respect and confidence of those who have achieved true eminence themselves, and value the like attainments in others. Do honor to your profession and to yourselves, or *desert* it entirely, for places of less fearful responsibility, and more lucrative returns. Dream not of *indolence* while hoping for *success* in the practice of medicine. It has for its votaries no syren song of ease. It promises only those pleasures which are felt when success has crowned the arduous, vigorous struggle. But for the faithful there are rewards which are worthy of the most vigorous efforts of your mature manhood.—To be satisfied with less, is to be unworthy of yourselves, of your age, and of your profession.

Do not think that I overrate the labors before you. I cannot, if I would.

The finger of Jehovah has never traced for you on tables of stone the great central truths of medical science, as it hath for the guidance of theologians and jurists. Nor hath He given upon the tablets of our hearts the unerring intuitions of medical practice. He has placed before us the human body, infinitely more complex, and perfect as a machine, than human genius can comprehend, mysteriously allied to a soul of which we know still less, and bid us study the anomaly of natural laws suspended, through the agency of vital power, developed by reason of such union.

Yours is the privilege, gentlemen, of associating your labor with those of some of the most noble and self-sacrificing spirits that ever have lived. Men who shrank not from plague nor pestilence, that they might learn its nature and mitigate its terrors; men, who like HAVEY were summoned to the courfs of kings as instructors; men, who like the youthful lamented BICHAT, spent their life-efforts in rooms so tainted by reason of experiments to determine the progress of decomposition of tissues, and in the study of morbid anatomy, that the very air they breathed was charged with fatal poison; men who have been the saviours of thousands upon the battle field, and in crowded hospitals, whose labors and hazards the world has too little known, and too little prized. But to be honorably associated with such, requires the aim and effort of men inspired with love for our profession, and a restless determination to excel. It requires that the *labor* be appreciated, and the *means* at once made use of which shall insure the desired result.

The advances in medical science have been attended with herculean labors.

To attain the present intimate knowledge of the human body; to understand the physiological actions of all its organs; to note the changes to which they are subject by reason of disease; to classify the great number of affections which we are called to treat; to comprehend the sanitary laws of mind and body; to acquaint ourselves with all the real therapeutic effects of the numerous and varied articles of the Materia Medica,—these were the labor of a life time—labor, from which one might shrink, were he not, by reason of such knowledge, to become of untold value to the world.

And yet this is but the real preparative work to an intelligent practice of the healing art; the knowledge with which you are to approach the bedside of the patient, and commence the practice of medicine.

I do not say that such *are* the attainments of all who assume such positions and such responsibilities. It is a matter of deep regret—nay, of grief—to the educated and conscientious, that, in so many instances, the facts are far otherwise, and that by the three-fold influence of ignorant practitioners, of cunning quacks, and money scheming patent-venders, the profession is brought into unmerited disgrace.

Many, indeed, have been the errors of visionists and theorists of honest intention, and our art has suffered in its onward progress by reason of them.

The Bible should have settled all disputes upon matters of theology if men would think alike; and the Golden Rule have governed the decisions of jurists if justice were the same idea in every mind; but, despite that heaven-given revelation, divines and would-be divines do disagree; and among the ermine-clad are found strangely conflicting rulings. But I am not to doubt the truths of theology, nor am I to discard the courts.

There is a pure theology—there can be but one. There are eternal truths for the guidance of jurists, in conflict with which they should never attempt to rule, knowing their decisions must be reversed. There is but a single science of medicine—there can be but one. Man has certain relations to his surroundings, and they are the same whoever may study them. The influences that injure and which benefit him are fixed. The means of benefiting him, and of modifying or removing his ill, are not conflicting. We may sometimes vary our efforts for accomplishing a desired result, as the jurist may, by one illustration or another, expound the law; but the result sought is one and the same—recovery.

When in theology I find strange discrepancy in teachings, I betake myself not alone to the word of inspiration for my conclusions of right. I confess my inability to grasp, at the moment, the abtruse points at issue, and I hesitate to trust my judgment. I turn with reverence to those who have been of undoubted ability, of unquestioned piety, and whose teachings seem but the elucidations of divine truth. If for successive ages the great and good accept and teach a similar faith, I accept that docrine as safe for me, and to listen to such teachings I would ever frequent the house of God.

I look with suspicion upon all those placards and posters which announce the arrival of distinguished professors and titled men, who denounce the wisdom of the old divines as foolishness, and promise to show men a more reasonable and free way of salvation, and only charge ten cents for admission.

So, too, when there are discrepancies in the rulings of the courts, I profess not always to be able to balance the rights at issue, of belligerents, but I seek the opinions of men of known ability, of spotless integrity, in positions free from any possibility of bias, and if I find, for the successive ages, the rulings of such men have been in the main in harmony, I am influenced by their views and receive my opinion as the result of their united wisdom.

I think eminent jurists have never been known to publish their own greatness, or the number and correctness of their rulings. The bare-faced act would brand the villain as a legal quack, and from such disgrace the legal profession is exempt.

By such rule it would be our pride and pleasure, that men should judge of us. It cannot be that the carefully observed facts of most eminent men should be of no importance; that there should be no gradually developing but permanent system of medicine. It cannot be that we are to exercise less self-respect, and boast of our abilities on posted handbills, and by displayed advertisements. How long shall such unmasked quackery be tolerated and patronized in an intelligent and sensible community?

How long shall confident boasters of success and braggart villains, with specious promises of cure, deceive the unfortunate and degrade the profession?

Till such a time as sensible men shall use their influence in a becoming manner with reference to the patronage of these deceiving scoundrels.

And how long, too, shall the ready assurance of many of our fair ones declare to us the method of our practice?

It is to me a matter of surprise that many of our ladies of good intention, perhaps, should so readily assume an ability to treat disease to which our wisest practitioners have not attained.

As ignorant of the human system and its necessities as they are of the contents of a Chinese puzzle-box, if they would only confess what sensible men all know, they are prompt, and in many instances officious and gratuitous, in passing upon the relative merits of the so-called systems of practice; are never in doubt as to the needed medication in a given disease; and pass upon the relative abilities of medical men with a dispatch and nonchalence, which put modesty and ordinary ability quite to the blush.

I speak not thus of all. I only allude to that well-meaning, but evil-doing brood in our communities, which may be termed medical busy-bodies, flattered by a certain class of practitioners for the train of influences which follow in their pathway.

If their delicate potations and aromas do no good, how do they know they will do no hurt? If the chimney simply be burning out, their labors of love may be all right, but, if the house be really on fire, while their delicate fingers gather pearly dewdrops from beautiful flowers, and dampen the doorsteps with the pure crystal waters from heaven, which surely can do no harm, that house may burn down, and amid its ruins may smoulder the form of the dearest idle around which their affections shall ever twine.

The taste of the child may govern the whim of the mother. The mother controls the father's purse, and that purse, if it is a long one, always procures the sugar prescription; and, if nature is adequate to the work, it may all be well enough. But, when the hour of sad anguish is at hand, if there be any truth and value in the testimony of worthy men and active remedies, that family reckons without its medical aid.

Do not deem me disturbed for the permanence of medical science, or the successful career of its worthy practitioners, by what I have said. I think the matter of a medical fee for us can hardly equal in importance the health and happiness of the one whose life is imperiled, and which with regret we behold trifled with, or needlessly thrown away.

Nor would I counsel a course of treatment of unnecessary severity. I more deprecate the aimless dosing treatment, than that to which I have already referred. There have been errors in medical practice—gross ones, too. So divines burned witches two hundred years ago,—but the wiser ones have abandoned that practice some time since; and gifted and skillful men have been prudent and cautious in the use of active medicines—frank to undeceive those who were not in need, and gradually leading their patients to an intelligent care of themselves, without the expense and deception to which another class are constantly subject.

The time was when the practice of medicine was greatly overrated, and all cures were the veritable results of wise medication, as the people believed. Strange and diverse are the teachings and practice of the healing art in these latter days. And, since there are recoveries under all forms of treatment, the community are driven to the nearly opposite extreme, and are skeptical as to the benefits of medicines at all.

But the course of medical instruction is daily more simple, more practical, and in its results more beneficial to mankind.

The necessity of thorough education is more and more apparent in nearly all our schools; the facilities of instruction are everywhere being extended; and I may simply add that, for the purpose of testing a method of medical teaching which should increase still more the student's privileges, this school had its origin.

I have only alluded to your surroundings, not as discouragements, but that you should so labor to fit yourselves by the means at hand as to render broad and marked the distinction between your course as professional gentlemen, and the noisy and unscrupulous impostors with whom you will be in contact.

If you shall keep pace with the times, your labors are only now commenced. No profession can boast of abler or more industrious men than to-day, in every civilized nation, are freely contributing their life-labors to the advancement of medical science. Our journals are replete with discoveries and valuable instructions. Not a nation, but may be proud of its teachers and its writers, whose reputation and discoveries we claim as a common heritage.

Here, at the outset of your career, if you dare to assume a mission so noble and responsibilities so fearful, pledge in honor your vigorous efforts to attain that knowledge of your profession which shall in the future render you its worthy representatives.

Gentlemen, hitherto I have addressed you as students; I hail you now as brothers. Months of intimate relation have changed a casual acquaintance, or the meeting of strangers, for this, the parting of intimate friends.

You will bear witness to the earnest efforts of the Faculty in every possible manner to render the time of your sojourn with us of the greatest possible profit to yourselves.

Especially in the clinical department has this been eminently true, and I know that you appreciate the fact.

You have the accredited testimonials of this institution as a just tribute of our esteem for you and for your professional attainments.

Guard well the honor of your Alma Mater, and give her in the profession, in your persons, a representation of which she may be justly proud.

Strive each of you to render life a noble success. To your natural qualifications add the cultivation of intellect, of morals, and of manners, which shall, to the best of your ability, fit you for your noble mission.

How precious are the jewels yet to be committed to your guardianship! How saddened hearts will thrill or cease to beat, as the weal or woe of the sick are graven upon your features, even in the still room when death seems near!

If then and there you comprehend the sufferer's want, and can seize the remedy, familiar perhaps to all, but whose virtue none may know, and by your discrimination, so apply it that the exhausted powers of nature shall feel its aid, and, rallying, restore the system to its former health, God never gave to man but a single nobler deed to do, and that, to point that spirit to Himself!

It is needless to add that your moral integrity should be beyond the possibility of question.

Every claim of your profession, every relation with the community, every interest of your own, point to a spotless life and a pure heart as your peculiar requisite.

Degrade not yourselves nor your calling by impurity of language, of habit—nay, even of thought.

Shun all profanity; shun the intoxicating bowl as a deadliest enemy; and shun, too, those disgusting personal habits which render not a few physicians so objectionable in the sick room.

Let your warm-hearted philanthropy render your ever alive to the sufferings of your fellow-beings. It should lead you to the lone and humble cottage, if there dwell the children of want, though no pecuniary influence should tempt you there. The satisfaction which arises from the consciousness of benefiting a fellow-being, or alleviating his sufferings and healing his maladies, shall in the future be a reward unequaled by gold. The gratitude of the poor shall centre on you, and the blessings of a philanthropist shall crown your lives.

You should be men of prompt decision. From the very nature of your profession, where the stout heart fails and the strong arm trembles, there should your hand be steady and your firmness manifest. The saving of a life may be the work of a moment, but that act may require the quick discernment, the mature judgment, and the intrepid coolness which well might covet a longer time for their proper exhibit.

Trespass not, in the most delicate manner, upon the rules of etiquette with your professional brethren. Stand gently, firmly, manfully, for your rights as medical men in every respect, and as firmly and as manfully regard and maintain the rights of your associates. Be open-hearted, frank, courtous, in all your professional relations: and from such as abuse such generous confidence, quietly withdraw yourselves, and seek not their fellowship, for such are dangerous men. But, when professional worth is found, wed its weal and fortune to yourselves as with hooks of steel.

To increase your efficiency and usefulness, you should be the diligent students of human nature. That knowledge should prepare you for every possible vicissitude. Called to act among all classes of society, your dignified yet affable deportment should commend you to the poor, while your general knowledge and careful observance of the rules of etiquette should render you at once the welcome and profitable companion of the affluent and cultivated.

Let me add, in conclusion, you will only, in the highest sense, meet the requirements of your mission when, with devout reverence you recognize in yourselves the simple instruments in the hands of Him, with whom are the issues of life, in the accomplishment of His merciful purposes to a suffering humanity. And, when all that men may do, has been accomplished, it may be to you the consolation which every medical man needs in his constant grapples with the King of Terrors, to commit the issue of the struggle to Him who dealeth gently, and who doeth all things well.

To-night there is a severance of our former pleasing relations. You turn from us to greet the loved ones at home, and practice the profession of your choice. Your journey will be along the banks of that cold stream, into the darkness of which, fondest friends are constantly passing, but from which none ever return.

Upon the banks of the river of death you shall pitch your ever-moving tents as wreckers and rescuers. The unseen power that draws to that shore the infant and the aged, you shall sometimes successfully breast; snatch the victim from the Lethean tide; brush the tears from weeping eyes, and bid the sorrowing bear back their precious ones, with joyous steps and happy hearts, to life and home again.

Anon, the mother, like the willow bent to earth, shall bring her first-born, her darling child, quite to the river's edge, and wildly wish to reach beyond and grasp again the parted hand. When she turns to you with piteous prayer, "Oh, Doctor, save my child!" then will you feel, as you never felt before, the feebleness of human agencies, and you can only say, God's will, sad heart! God's will be done.

From the high and rugged cliffs above you, with one fell plunge, the mad inebriate, the suicide, and the assassin's victim shall sink beneath the dark wave, with never a rescuing hand for their recovery.

At his appointed time the aged man shall come—green the velvet lawn upon which he treads; gentle the banks' descent; kind friends attend; calm and peacefully he gathers his vesture around him, and sinks so gently to his rest that your experienced eye scarce notes the moment when his frail bark had parted for the other side. Peaceful and happy, it was his time; he had no need of you. You had no healing balm for him.

Thus shall you journey on. Upon the one hand are the agonies, the groans and tears of the sorrowing; and upon the other, the darkness and stillness of that mysterious river.

To thousands of hearts you shall be as angels of mercy—often to save, always to comfort.

Prize, then, the privilege of such noble deeds, and, in a calm old age, may yours be a peaceful transit to the shining shore where our vocation is unknown.

Till then, go forth, and quit yourselves like men!

Till then, go forth, and ever—FARE-YOU-WELL.

ARTICLE XII.

